# Cluster of Norovirus Genogroup IX Outbreaks in Long-Term Care Facilities, Utah, USA, 2021

**DOI:** 10.3201/eid2811.220842

**Published:** 2022-11

**Authors:** BreAnne Osborn, Chao-Yang Pan, April Hatada, Jennifer Hatfield, Jenni Wagner, Kelly Oakeson, Anna Montmayeur, Christina Morales, Jan Vinjé

**Affiliations:** Utah Department of Health, Salt Lake City, Utah, USA (B. Osborn, J. Wagner, K. Oakeson);; California Department of Public Health, Richmond, California, USA (C.-Y. Pan, A. Hatada, C. Morales);; Utah County Health Department, Provo, Utah, USA (J. Hatfield);; Centers for Disease Control and Prevention, Atlanta, Georgia, USA (A. Montmayeur, J. Vinjé)

**Keywords:** Norovirus, enteric infections, food safety, viruses, acute gastroenteritis, long-term care facilities, Utah, United States

## Abstract

We report 5 clustered acute gastroenteritis outbreaks in long-term care facilities in Utah, USA, that were linked to healthcare employees working at multiple facilities. Four outbreaks were caused by norovirus genotype GIX. We recommend continued norovirus surveillance and genotyping to determine contributions of this genotype to norovirus outbreaks.

Norovirus is the leading cause of acute gastroenteritis worldwide (*1*). The virus can be transmitted through person-to-person contact, aerosolized vomitus, contaminated food or water, or fomites (*2*). Noroviruses are divided into 10 genogroups; viruses in genogroups GI, GII, GIV, GVIII, and GIX cause illness in humans. Norovirus GIX was first identified in fecal samples collected in 1990 from US troops deployed to Saudi Arabia (*3*). This genogroup was previously known as GII.15 and was reclassified recently (*4*).

Although global norovirus surveillance is limited, several studies have attempted to quantify the prevalence of norovirus genotypes. In the United States, >99% of all norovirus outbreaks are caused by GI and GII viruses (*5*); most outbreaks are associated with GII.4 Sydney (*4*). Globally, norovirus GIX has been detected less frequently and has not been associated historically with large outbreaks (*5*–*10*). During 2009–2016, two norovirus GIX outbreaks were reported to CaliciNet, the US norovirus surveillance network (*5*,*10*). Similarly, during 2016–2018, only 1 of 556 norovirus outbreaks reported to China’s norovirus outbreak surveillance network was associated with norovirus GIX (*6*). We describe a cluster of 4 epidemiologically linked norovirus GIX outbreaks and 1 suspected GIX outbreak among long-term care facilities (LTCFs) in Utah during 2021.

## The Study

On March 31, 2021, the Utah County Health Department and Utah Department of Health were notified of an outbreak of gastrointestinal illness at LTCF A. The outbreak was believed to have originated from 2 residents on March 28 and 29. One resident vomited in a well-trafficked, carpeted hallway, which likely contaminated the environment. By mid-April, 4 other LTCFs (B–E) within 20 miles of facility A reported similar outbreaks.

We asked LTCFs to provide data on resident and staff illnesses and a list of residents who were receiving services from home healthcare companies. We conducted interviews with home healthcare employees in September 2021 to identify symptoms of gastrointestinal illness, which residents were cared for by those employees, and which facilities they worked in.

We collected fecal samples from symptomatic residents and staff at facilities A–D during active illness; no samples were collected from facility E. After etiology was confirmed as norovirus by the Utah Public Health Laboratory, we forwarded all samples to the California Department of Public Health Viral and Rickettsial Disease Laboratory, which serves as a CaliciNet outbreak support center for genotyping and next-generation sequencing.

We extracted nucleic acids from fecal specimens using the NucliSENS easyMAG instrument (bioMérieux, https://www.biomerieux.com) and genotyped norovirus-positive samples by using conventional reverse transcription PCR (*11*). We submitted purified PCR products to Sequetech (https://www.sequetech.com) for Sanger sequencing and genotyped by using the human calicivirus typing tool (https://calicivirustypingtool.cdc.gov) (*12*). We further analyzed norovirus-positive samples by performing next-generation sequencing (NGS) of complete genomes (*13*) using the Illumina MiSeq platform (Illumina, https://www.illumina.com) and a GIX-specific forward oligonucleotide primer (5′-ATGGCGTCGARTGACGTCGYTACTGCCYTTGGC-3′). We analyzed sequences by using the Viral NGS Analysis Pipeline and Data Management tool (*14*). We generated norovirus phylogenetic trees for complete RNA-dependent RNA polymerase (*RdRp*) (1,430 nt) and major capsid (1,668 nt) genes by using MEGA11 software (*15*).

**Figure 1:**
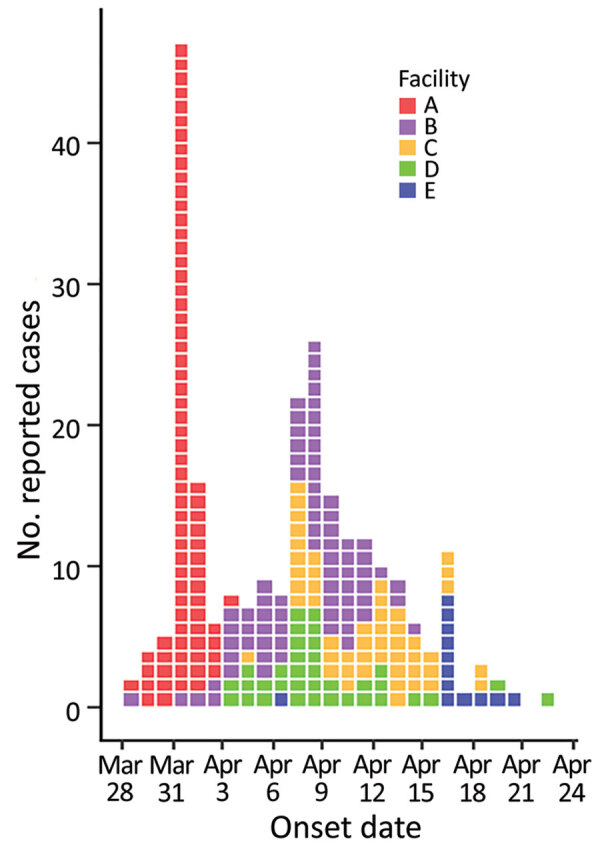
Onset dates for reported cases of acute gastroenteritis among 5 long-term care facilities during March 28–April 24, 2021, in study of cluster of norovirus genogroup IX outbreaks in long-term care facilities, Utah, USA, 2021. Of 290 total reported cases of acute gastroenteritis, we were able to obtain onset dates for 247 cases. Each colored box represents 1 case of acute gastroenteritis.

Among the 5 LTCFs, 290 persons reported gastrointestinal symptoms: 39/74 (53%) residents and 43 (of an unknown total) staff in facility A, 47/68 (69%) residents and 30/66 (45%) staff in facility B, 32/58 (55%) residents and 20/75 (27%) staff in facility C, 37/97 (38%) residents and 29/85 (34%) staff in facility D, and 5/100 (5%) residents and 8/85 (10%) staff in facility E (Figure 1). In addition, 5/10 (50%) home healthcare employees reported they were ill; 2 employees worked in facilities A and B, 1 worked in facilities A and C, and 2 worked in facilities A and D.

A total of 14 fecal samples were collected: 6 samples from residents in facility A, 2 samples each from residents in facilities B and C, 3 samples from residents in facility D, and 1 sample from a home healthcare employee who worked in facilities A and B. Of those samples, 13 (93%) tested positive for norovirus; 1 sample from facility D was negative. Although the home healthcare employee’s sample was norovirus-positive, the virus could not be genotyped.

**Figure 2:**
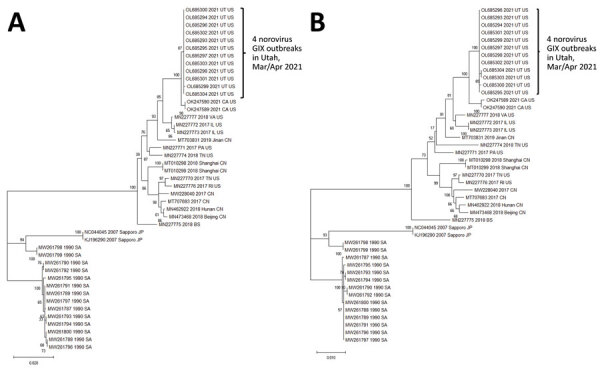
Phylogenetic comparisons of norovirus genes in study of cluster of norovirus genogroup IX outbreaks in long-term care facilities, Utah, USA, 2021. We generated phylogenetic trees by using the maximum-likelihood method and Tamura-Nei distance model (*15*). We compared nucleotide sequences of the *RdRp* gene (1,430 nt) (A) and major capsid gene (1,668 nt) (B) from the 12 sequences obtained from the 4 LTCF outbreaks with 33 GIX strains obtained from GenBank. The bootstrap percentages are shown next to the branches. We generated initial trees automatically by applying neighbor-joining algorithms to a matrix of pairwise distances estimated by using the maximum composite-likelihood approach and then selecting the topology with the superior log-likelihood value. We conducted evolutionary analyses by using MEGA11 software (*15*). Scale bars indicate nucleotide substitutions per site.

We obtained partial sequences of *RdRp* and capsid genes from 12 of 13 positive specimens by using dual region reverse transcription PCR, genotyped the virus as norovirus GIX.1[GII.P15], and uploaded the sequence data into the CaliciNet database. All 12 partial *RdRp* or capsid sequences showed 100% nucleotide identity. NGS produced near-complete genomes (≈7,490 nt) for all 12 specimens, which were 99.9%–100% identical. The closest matching sequence in GenBank (accession no. MN227777) had a 98% nucleotide identity. By using phylogenetic comparisons of complete *RdRp* and capsid nucleotide sequences (Figure 2), we determined the 12 sequences from facilities A–D were closely related to LTCF outbreaks in California in 2021 (GenBank accession nos. OK247589 and OK247590). We submitted the near-complete genomic sequences for the 12 specimens from Utah to the National Center for Biotechnology Information (accession nos. OL685293–304).

We determined that the same home healthcare company provided services to residents in 4 of the outbreak facilities (A–D). A norovirus-positive fecal sample was collected from a resident of facility A who received care from home healthcare employees who also reported they had acute gastroenteritis symptoms. Home healthcare services were received by 2 other residents of facility A who became ill. In addition, the earliest onsets of illness were observed in residents of facilities B and C who received care from the same home healthcare company. Facility E reported some of their residents had received services from the same home healthcare company, but not enough information was available to establish a definitive epidemiologic link. All but 1 home healthcare employee who reported illness worked in a facility that experienced an outbreak.

## Conclusions

We report the relatively rare norovirus GIX as the cause of 4 LTCF outbreaks in Utah during March–April 2021. Epidemiologic evidence and sequencing of norovirus genomes suggested the outbreaks in facilities A–D were related, likely transmitted through employees of a home healthcare company. Although available laboratory and epidemiologic data do not definitively connect the outbreak in facility E with outbreaks in facilities A–D, we suspect a connection exists because of similarities in temporal, geographic, symptom, and setting characteristics of the outbreaks.

Our investigation highlights the ability of norovirus to spread rapidly despite increased disease prevention measures established during the COVID-19 pandemic. Whereas some pandemic restrictions were beginning to ease in the spring of 2021, LTCFs in Utah maintained precautions, including enhanced cleaning protocols. In addition, the home healthcare company that provided services to the facilities in our investigation reported limiting the number of facilities where each employee worked to prevent COVID-19 transmission between facilities. Our results show that these precautions were insufficient to prevent transmission of norovirus GIX and emphasize the overall challenges of controlling norovirus outbreaks.

In addition to these outbreaks in Utah, norovirus GIX was reported as the cause of 7 acute gastroenteritis outbreaks in other states during September 1, 2020–September 30, 2021 (https://www.cdc.gov/norovirus/reporting/calicinet/data.html). These numbers represent a substantial increase in reported GIX outbreaks in the United States, considering only 2 were reported during 2009–2016 (*5*,*10*). We recommend continued norovirus surveillance and genotyping to determine contributions of the uncommon GIX genotype to increasing norovirus outbreaks.
